# Factors Affecting the Choice of Neurosurgery as a Future Career: A Cross-Sectional Study

**DOI:** 10.7759/cureus.52836

**Published:** 2024-01-23

**Authors:** Farooq A Wani, Khalid M Alanazi, Abdalrhman S Alblwan

**Affiliations:** 1 Pathology and Laboratory Medicine, Jouf University, Sakaka, SAU; 2 Medicine and Surgery, Jouf University, Sakaka, SAU

**Keywords:** factors, career option, medical school students, surgical specialty, neurosurgery

## Abstract

Introduction

Selecting a specialty is a very important and stressful decision that students must make, as it will have a lasting impact on their professional lives. Medical students could gain insight into a variety of specialties during their clinical years, especially the work environment in different specialties. Numerous factors can influence this decision, such as work-life balance, lifestyle, and gender differences. The goal of our study is to demonstrate the different factors, both attractive and deterrent, that influence neurosurgery selection as a future specialty among students. Also, we will consider the exposure-related geographical distribution of the neurosurgery field regarding conferences and workshops, as well as the availability of university professors and their impact on the interest of students in the specialty.

Methods

A cross-sectional study spanning from June 2023 to September 2023 was conducted among students at medical colleges across the Kingdom of Saudi Arabia. All medical students from the second medical year up to the internship were invited to take part in the study. Non-medical students, first-year medical students, and incomplete questionnaires were excluded. Employing a stratified random sampling technique, we ensured diverse representation, eventually gathering data from 1141 participants.

Results

The study involved a diverse group of 1141 medical students and interns, with an average age of 21.7 years. Among them, 683 (59.9%) were female. Approximately half of the participants, 572 (50.1%), express an interest in pursuing a career in neurosurgery, and a significant portion of respondents find neurosurgery appealing due to its challenging nature (50.9%). On the other hand, stress emerged as the most significant deterrent factor (50.3%). Among educational levels, interns, 39 (28.3%), showed the least interest, while second-year students, 193 (64.8%), exhibited the highest interest (p < 0.001*). The analysis revealed statistically significant gender differences in factors. Specifically, a higher percentage of females found "interested in neuroscience" to be the most attractive factor compared to males (18.2% vs. 15.5%, p < 0.001*). Regarding deterring factors, a greater percentage of males found "risk" as the most deterring factor compared to females (19.2% vs. 17.1%, p = 0.001*).

Conclusion

We found no significant age variation in the most attractive factors; however, significant gender differences in attractive and deterrent factors were observed. The "risk" associated with neurosurgery was the most deterring factor for students across different cumulative grade point average (CGPA) ranges and for students from the eastern and central areas. Our findings suggest that most factors are consistently attractive or deterring across different educational and clinical levels, emphasizing the stability of these perceptions throughout medical education. We propose innovative educational initiatives with increased faculty participation to implement the curricula with early exposure of students to neurosurgery.

## Introduction

As a recognized specialty, neurosurgery is one of the most recent and quickly expanding surgical fields. The field of neurosurgery is based on the diagnostic and treatment aspects of illnesses of the brain, spine, spinal cord, and peripheral nerves. While being primarily a surgical field, neurosurgery necessitates an understanding of neurology, critical care, trauma care, and imaging [1]. Selecting a specialty is a very important and stressful decision that students must make, as it will have a lasting impact on their professional lives. Medical students could gain insight into a variety of specialties during their clinical years, especially the work environment in different specialties. This can help them decide whether they would like to pursue, become interested in a specific specialty, or choose a different career path [2]. Numerous factors can influence this decision, such as work-life balance, lifestyle, and gender differences. Even with these considerations, students usually find ways to make informed decisions throughout their medical school years, especially clinical years and rotations, by having exposure to multiple specialties and learning about the pros and cons of each one [3].

Based on a World Health Organization study published in 2019, there are 49,940 neurosurgeons across the globe [4]. In Saudi Arabia, the number is much lower, with only 238 neurosurgeons, according to research conducted in 2018 [5]. There is one neurosurgeon per 250,000 people in the Kingdom of Saudi Arabia (KSA), and there has been speculation that the paucity of neurosurgeons is due to the limited opportunities for neurosurgical training abroad. An expanding population necessitated the development of a national neurosurgery training program [6]. Various non-modifiable factors, such as gender disparities, can affect students' decisions to pursue the neurosurgery field. Females are less likely to pursue this path [7]. On the other hand, factors that can be influenced during medical school or an internship, such as life-work balance, lifestyle, stress levels, personal preference, and long working hours, can also shape one's decision [8]. Early exposure to neurosurgery has been shown to influence students' perceptions of the specialty and impact their applications to it. Medical students who were exposed to neurosurgery early increased their applications for neurosurgical positions [9]. Neurosurgery residents encounter a distinctive set of challenges, including a huge workload, a long residency program, and unpredictable employment prospects. Long working hours extending beyond 80 hours per week and the intimidation of residency training in general are other challenges [10].

Inherent interest in neurosurgery, encouragement from seniors and mentors during clinical postings, and the glamour of the specialty were the most frequent reasons given for pursuing the specialty. Several other motivations include familial inspiration, additional research opportunities, and a passion for a delicate specialty requiring meticulousness [11]. Neurosurgery is among the most competitive residencies, attracting only the finest and brightest applicants from reputable medical schools. As a result, most students admitted to a neurosurgical training program are exceptional, being among the toppers in their class and having higher percentiles [1]. Previous studies have been conducted in some areas of Saudi Arabia, but none have covered the whole Kingdom of Saudi Arabia [6,12]. Hence, the goal of our study is to demonstrate the different factors, both attractive and deterrent, that influence neurosurgery selection as a future specialty among students. Also, we will consider the exposure-related geographical distribution of the neurosurgery field regarding conferences and workshops, as well as the availability of university professors and their impact on the interest of students in the specialty.

## Materials and methods

Study design

A prospective cross-sectional study spanning from June 2023 to September 2023 was conducted among students at medical colleges across the Kingdom of Saudi Arabia. The medical colleges belonged to Jouf University, Taibah University, Majmaah University, King Abdulaziz University, King Khalid University, King Faisal University, King Saud bin Abdulaziz University for Health Sciences, Taif University, Tabuk University, Alfaisal University, Imam Abdulrahman Bin Faisal University, Princess Nourah bint Abdulrahman University, and Bisha University. Other medical colleges included Batterjee Medical College, Alrayan Medical College, and Vision Medical College.

Inclusion criteria

All medical students from the second medical year up to the internship were invited to take part in the study.

Exclusion criteria

We excluded non-medical students, first-year medical students, and incompletely filled questionnaires.

Employing a stratified random sampling technique, we ensured diverse representation, eventually gathering data from 1141 participants. Data collection utilized a structured online questionnaire offered in English and distributed via Google Forms. We followed CHERRIES guidelines while preparing the Google Forms, which included the purpose of the study, the number of questions, the time needed to finish the survey, the risks and benefits associated with the survey, and the confidentiality of the data. A statement of consent followed. This tool is completed within three to five minutes and comprises four distinct sections. The first section is regarding data-capturing aspects like age, gender, education level, grade point average (GPA), and geographic location. The second section was regarding participants’ interest and knowledge of neurosurgery as a career, what is required of them to get accepted and enrolled in the residency program, and whether they attended a course, conference, or have done a neurosurgery-related project. The third section was about attractive and deterrent factors in neurosurgery. A lot of factors were asked to know what attracts the students toward the specialty, including that it is a challenging specialty, their interest in practical aspects, surgical techniques or high skills, income, impact on patients, and other factors. Among deterrent factors, students were also asked about stress, long training time, work-life balance, and risk associated with the specialty.

A preliminary pilot study was executed to gauge the questionnaire's completion time, excluding its participants from the primary study. To ensure ethical research practices, we obtained ethical approval from the Local Committee of Bioethics (LCBE) of Jouf University, vide approval no. 2-10-44, dated June 19, 2023, and ensured participants' informed consent.

Statistical analysis

We performed descriptive and inferential analyses. Frequencies and percentages were calculated and tabulated for categorical variables. Means and standard deviations were used as measures of central tendency and dispersion, respectively, for continuous variables owing to the large sample size and relatively normal distribution of the variables assessed by Q-Q plots. Additionally, the data was visualized where possible for easier interpretation.

To find out the factors associated with interest in neurosurgery as a career, Fischer’s exact test was applied and interpreted for categorical variables and an independent sample t-test for continuous variables. Furthermore, to predict the odds of interest in neurosurgery, a binary logistic regression model was created with the factors and other variables of interest included as predictors. A p-value of 0.05 or less, indicating a 95% confidence interval, was considered significant. IBM SPSS Statistics for Windows, Version 27.0.1 (released 2020; IBM Corp., Armonk, New York, United States) was used for statistical calculations.

## Results

Table 1 shows the characteristics of the participants. The study involved a diverse group of 1141 medical students and interns, with an average age of 21.7 years. Among them, 683 (59.9%) were female, and 458 (40.1%) were male. The participants spanned various academic years, with the majority in the second, 298 (26.1%), followed by the third, 229 (20.1%), and the fourth, 205 (18.0%) years. Additionally, 409 (35.8%) were in clinical years (fifth, sixth, internship), while 732 (64.2%) were in pre-clinical years (second, third, fourth). Academic GPA ranged from below 2.5 (1.2%) to 4.75-5 (28.1%). Geographically, the participants represented different regions, with 316 (27.7%) from the central region, 280 (24.5%) from the northern region, 216 (18.9%) from the western region, 191 (16.7%) from the eastern region, and 138 (12.1%) from the southern region.

**Table 1:** Sociodemographic characteristics of the participants GPA: grade point average

		Mean	Standard deviation	Count	Percentage %
Age		21.7	2.1		
Gender	Female			683	59.9%
	Male			458	40.1%
Educational level	Second year			298	26.1%
	Third year			229	20.1%
	Fourth-year			205	18.0%
	Fifth year			140	12.3%
	Sixth year			131	11.5%
	Internship			138	12.1%
Educational level categories	Pre-clinical (2nd, 3rd, 4th Year)			732	64.2%
	Clinical (5th, 6th, Internship)			409	35.8%
Academic GPA	<2.5			14	1.2%
	2.5-2.99			34	3.0%
	3.0-3.49			78	6.8%
	3.5 – 3.99			196	17.2%
	4.0 – 4.49			277	24.3%
	4.5-4.74			221	19.4%
	4.75 – 5			321	28.1%
Region	Central			316	27.7%
	Eastern			191	16.7%
	Northern			280	24.5%
	Southern			138	12.1%
	Western			216	18.9%

Table 2 shows the interest of the participants in neurosurgery. Approximately half of the participants, 572 (50.1%), express an interest in pursuing a career in neurosurgery, and a majority, 762 (66.8%), claim to understand what such a career entails. However, a lower percentage of 507 (44.4%) feel confident in their understanding of the requirements to secure a training position in neurosurgery. Notably, a substantial portion of 523 (45.8%) have received neurosurgical teaching during their medical education. In terms of exposure, 359 (31.5%) participants have previously attended a neurosurgical conference or course, and an even smaller fraction, 277 (24.3%), have completed additional degrees, student-selected components, or projects related to neurosurgery. These findings provide insights into the participants' levels of interest and prior exposure to the field of neurosurgery.

**Table 2:** Interest of participants in neurosurgery

Question	Response			
	No		Yes	
	Count	Percentage %	Count	Percentage %
I am interested in a career in neurosurgery	569	49.9%	572	50.1%
I understand what a career in neurosurgery involves	379	33.2%	762	66.8%
I understand what is required of me to obtain a training number in neurosurgery	634	55.6%	507	44.4%
I have previously attended a neurosurgical conference/course	782	68.5%	359	31.5%
I have completed an additional degree, student-selected component, or project related to neurosurgery	864	75.7%	277	24.3%
I have received neurosurgical teaching during my medical education	618	54.2%	523	45.8%

**Figure 1:**
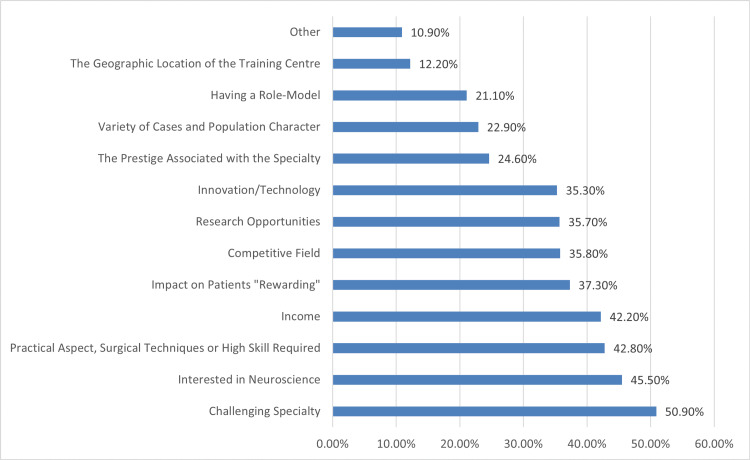
Factors attractive to neurosurgery

Figure 1 shows factors attractive to neurosurgery. A significant portion of respondents find neurosurgery appealing due to its challenging nature (50.9%) and showed their interest in it (45.5%). Additionally, the practical aspects and high skill requirements of the specialty (42.8%), income potential (42.2%), and rewarding impact on patients (37.3%) are cited as attractive aspects. Furthermore, the competitive nature of the field (35.8%) and research opportunities (35.7%) are notable factors.

**Figure 2:**
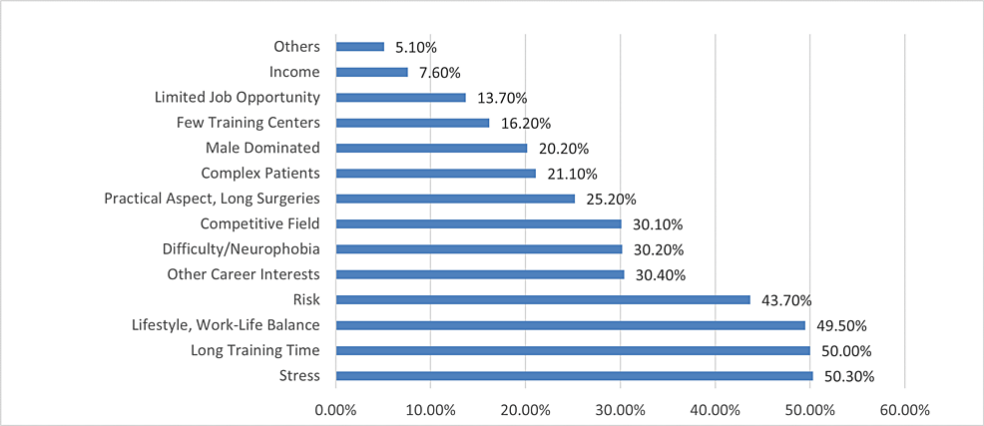
Factors deterring neurosurgery

Figure 2 shows factors deterring neurosurgery. Stress emerged as the most significant deterrent (50.3%), followed closely by the long training duration (50.0%) and concerns related to lifestyle and work-life balance (49.5%). Participants also expressed reservations about the perceived risk associated with neurosurgery (43.7%) and competing career interests (30.4%). Factors such as the specialty's difficulty (30.2%) and the competitive nature of the field (30.1%) were also cited as deterrents.

Factors predicting the choice of neurosurgery as a career

Univariate Analysis

Notably, several factors were significantly correlated with an interest in neurosurgery. Among educational levels, interns, 39 (28.3%), showed the least interest, while second-year students, 193 (64.8%), exhibited the highest interest (p < 0.001*). Similarly, participants in the clinical phase, 155 (37.9%), were less interested compared to those in the pre-clinical phase, 417 (57.0%) (p < 0.001*). Academic GPA played a significant role, with lower GPAs associated with greater interest (p < 0.001*). Understanding the nature of a neurosurgery career (425, 55.8%), the requirements for obtaining a training number (286, 56.4%), attending neurosurgical conferences/courses (227, 63.2%), completing neurosurgery-related projects (180, 65.0%), and receiving neurosurgical teaching (279, 53.3%) were all associated with increased interest (p < 0.001*, p < 0.001*, p < 0.001*, p < 0.001*, p = 0.050*, respectively). Additionally, age differed significantly, with those interested in neurosurgery being slightly younger (mean age of 21.3 years) compared to those who were not interested (mean age of 22.1 years) (p < 0.001*). These findings underscore the influence of educational level, academic performance, and exposure to neurosurgical education on participants' interest in pursuing neurosurgery as a career (Table 3).

**Table 3:** Univariate analysis of factors associated with the choice of neurosurgery as a career GPA: grade point average

		I am interested in a career in neurosurgery				
		No		Yes		P value^F^
		Count	Row percentage %	Count	Row percentage %	
Gender	Female	328	48.0%	355	52.0%	0.131
	Male	241	52.6%	217	47.4%	
Educational level	2nd year	105	35.2%	193	64.8%	<0.001*
	3rd year	99	43.2%	130	56.8%	
	4th year	111	54.1%	94	45.9%	
	5th year	74	52.9%	66	47.1%	
	6th year	81	61.8%	50	38.2%	
	Internship	99	71.7%	39	28.3%	
Educational level categories	Pre-clinical (2nd, 3rd, 4th Year)	315	43.0%	417	57.0%	<0.001*
	Clinical (5th, 6th, Internship)	254	62.1%	155	37.9%	
Academic GPA	<2.5	3	21.4%	11	78.6%	<0.001*
	2.5-2.99	13	38.2%	21	61.8%	
	3.0-3.49	50	64.1%	28	35.9%	
	3.5 – 3.99	110	56.1%	86	43.9%	
	4.0 – 4.49	150	54.2%	127	45.8%	
	4.5-4.74	101	45.7%	120	54.3%	
	4.75 – 5	142	44.2%	179	55.8%	
Region	Central	170	53.8%	146	46.2%	0.244
	Eastern	91	47.6%	100	52.4%	
	Northern	126	45.0%	154	55.0%	
	Southern	70	50.7%	68	49.3%	
	Western	112	51.9%	104	48.1%	
I understand what a career in neurosurgery involves	No	232	61.2%	147	38.8%	<0.001*
	Yes	337	44.2%	425	55.8%	
I understand what is required of me to obtain a training number in neurosurgery	No	348	54.9%	286	45.1%	<0.001*
	Yes	221	43.6%	286	56.4%	
I have previously attended a neurosurgical conference/course	No	437	55.9%	345	44.1%	<0.001*
	Yes	132	36.8%	227	63.2%	
I have completed an additional degree, student-selected component, or project related to neurosurgery	No	472	54.6%	392	45.4%	<0.001*
	Yes	97	35.0%	180	65.0%	
I have received neurosurgical teaching during my medical education	No	325	52.6%	293	47.4%	0.050*
	Yes	244	46.7%	279	53.3%	
		Mean	SD	Mean	SD	P value^t^
Age (years)		22.1	2.1	21.3	2.1	<0.001
^F^Fischer's Exact Test ^t^Independent Samples t-test *p<0.05, Significant						

Multivariate Analysis

Several factors were found to be statistically significant predictors of interest in neurosurgery. Notably, students in the clinical phase of their education were about half as likely to express interest compared to their pre-clinical counterparts (p < 0.001*; adjusted odds ratio = 0.495). Regionally, participants from the northern region were more likely to be interested (p = 0.002*; adjusted odds ratio = 1.740). Additionally, several factors increased the likelihood of interest. For instance, understanding the nature of a neurosurgery career made participants about two times more likely to express interest (p < 0.001*; adjusted odds ratio = 1.975). Those who had previously attended neurosurgical conferences or courses were approximately 2.4 times more likely to be interested (p < 0.001*; adjusted odds ratio = 2.364), and individuals who had completed neurosurgery-related projects were roughly 1.8 times more likely to express interest (p = 0.001*; adjusted odds ratio = 1.817). These findings provide insights into the various factors that influence students' decisions to pursue a career in neurosurgery, including the educational phase, academic performance, regional factors, and exposure to neurosurgical education (Table 4).

**Table 4:** Multivariate binary logistic regression analysis of factors associated with the choice of neurosurgery as a career

Factors associated with interest in neurosurgery as a career		P value	Adjusted odds ratio	95% C.I. for AOR	
				Lower	Upper
Age (years)		<0.001*	.847	.778	.923
Gender	Female	Ref	Ref		
	Male	0.954	1.008	.770	1.320
Education level	Pre-clinical	Ref	Ref		
	Clinical	<0.001*	.495	.341	.718
Academic GPA	<2.5	Ref	Ref		
	2.5-2.99	0.708	.743	.157	3.520
	3.0-349	0.113	.314	.075	1.317
	3.5-3.99	0.306	.485	.121	1.942
	4.0-4.49	0.289	.475	.120	1.882
	4.5-4.74	0.546	.652	.163	2.608
	4.75-5	0.474	.604	.152	2.404
Region	Central	Ref	Ref		
	Eastern	0.141	1.344	.907	1.993
	Northern	0.002*	1.740	1.218	2.486
	Southern	0.254	1.303	.827	2.055
	Western	0.880	1.029	.707	1.498
Understanding what a career in neurosurgery involves		<0.001*	1.975	1.476	2.643
Understanding what is required to obtain a training number in neurosurgery		0.626	1.075	.803	1.441
Previously attended a neurosurgical conference/course		<0.001*	2.364	1.673	3.341
Completed an additional degree, student-selected component, or project related to neurosurgery		0.001*	1.817	1.265	2.609
Received neurosurgical teaching during my medical education		0.954	1.009	.748	1.361
*p<0.05, Significant					

Association of the most attractive and deterring factors with sociodemographics

Age

The mean ages of participants who found the field competitive, prestige associated with the specialty, rewarding impact on patients, income, research opportunities, interest in neuroscience, innovation or technology, geographic training location, challenging specialty, and practical aspects or surgical techniques most attractive ranged from 21.0 to 22.4 years, with no significant age-related differences (p = 0.077). Conversely, for the factors perceived as most deterring, participants who considered few training centers as the most deterring factor had a significantly higher mean age of 22.1 years compared to other factors (p = 0.003*). This analysis suggests that while most attractive factors showed no significant age variation, the perception of few training centers as a deterring factor was associated with older participants (Table 5).

**Table 5:** Association of the most attractive and deterring factors with age

		Age		
		Mean	Standard deviation	P value^A^
Most attractive factor	Competitive field	22.4	3.2	0.077
	The prestige associated with the specialty	22.2	1.9	
	Impact on patients “rewarding”	21.9	2.2	
	Income	21.8	2.2	
	Research opportunities	21.7	2.1	
	Interested in neuroscience	21.7	1.9	
	Innovation/technology	21.7	2.3	
	The geographic location of the training centers	21.6	1.6	
	Challenging specialty	21.5	1.9	
	Practical aspect, surgical techniques or high skill required	21.5	2.1	
	Others	21.4	1.8	
	Having a role-model	21.3	2.2	
	Variety of the cases and population character	21.0	1.7	
Most deterring factor	Few trainings centers	22.1	2.2	0.003*
	Competitive field	22.1	2.0	
	Long training time	22.0	1.9	
	Other career interests	22.0	2.4	
	Practical aspect, long surgeries	22.0	1.8	
	Income	21.8	2.2	
	Male dominated	21.8	2.4	
	Lifestyle, work-life balance	21.7	1.9	
	Risk	21.7	2.5	
	Stress	21.3	1.9	
	Difficulty/neurophobia	21.2	1.9	
	Complex patients	21.1	2.2	
	Others	20.7	2.1	
	Limited job opportunity	20.7	1.8	
^A^One-Way ANOVA *P<0.05, Significant				

Gender

The analysis revealed statistically significant gender differences in factors. Specifically, a higher percentage of females found "interested in neuroscience" to be the most attractive factor compared to males (18.2% vs. 15.5%, p < 0.001*). Regarding deterring factors, a greater percentage of males found "risk" as the most deterring factor compared to females (19.2% vs. 17.1%, p = 0.001*) (Table 6).

**Table 6:** Association of the most attractive and deterring factors with gender

		Gender				
		Female		Male		P value^F^
		n	Column percentage %	n	Column percentage %	
Most attractive factor	Interested in neuroscience	124	18.2%	71	15.5%	<0.001*
	Impact on patients “rewarding”	81	11.9%	62	13.5%	
	Challenging specialty	76	11.1%	56	12.2%	
	Practical aspect, surgical techniques or high skill required	88	12.9%	39	8.5%	
	Income	70	10.2%	54	11.8%	
	Innovation/technology	38	5.6%	51	11.1%	
	Competitive field	37	5.4%	40	8.7%	
	Research opportunities	45	6.6%	28	6.1%	
	Others	34	5.0%	20	4.4%	
	The prestige associated with the specialty	29	4.2%	13	2.8%	
	Having a role-model	32	4.7%	10	2.2%	
	Variety of the cases and population character	23	3.4%	7	1.5%	
	The geographic location of the training centers	6	0.9%	7	1.5%	
Most deterring factor	Risk	117	17.1%	88	19.2%	0.001*
	Lifestyle, work-life balance	125	18.3%	74	16.2%	
	Stress	79	11.6%	36	7.9%	
	Other career interests	68	10.0%	47	10.3%	
	Long training time	61	8.9%	54	11.8%	
	Competitive field	40	5.9%	47	10.3%	
	Difficulty/neurophobia	44	6.4%	29	6.3%	
	Male dominated	52	7.6%	14	3.1%	
	Others	22	3.2%	17	3.7%	
	Few trainings centers	27	4.0%	10	2.2%	
	Practical aspect, long surgeries	22	3.2%	12	2.6%	
	Complex patients	11	1.6%	18	3.9%	
	Limited job opportunity	9	1.3%	5	1.1%	
	Income	6	0.9%	7	1.5%	
^F^Fischer's Exact Test *P<0.05, Significant						

Academic CGPGA

Table 7 examines the association of the most attractive and deterring factors with students' academic CGPA. Several trends are notable.

**Table 7:** Association of the most attractive and deterring factors with academic CGPA CGPA: cumulative grade point average

		Academic GPA														
		<2.5		2.5-2.99		3.0-3.49		3.5 – 3.99		4.0 – 4.49		4.5-4.74		4.75 – 5		P value^F^
		n	Column percentage %	n	Column percentage %	n	Column percentage %	n	Column percentage %	n	Column percentage %	n	Column percentage %	n	Column percentage %	
Most attractive factor	Interested in neuroscience	4	28.6%	4	11.8%	14	17.9%	24	12.2%	48	17.3%	48	21.7%	53	16.5%	0.150
	Impact on patients “rewarding”	0	0.0%	2	5.9%	12	15.4%	18	9.2%	37	13.4%	32	14.5%	42	13.1%	
	Challenging specialty	1	7.1%	4	11.8%	6	7.7%	23	11.7%	43	15.5%	16	7.2%	39	12.1%	
	Practical aspect, surgical techniques or high skill required	2	14.3%	5	14.7%	9	11.5%	16	8.2%	28	10.1%	28	12.7%	39	12.1%	
	Income	0	0.0%	2	5.9%	8	10.3%	23	11.7%	31	11.2%	20	9.0%	40	12.5%	
	Innovation/technology	1	7.1%	4	11.8%	7	9.0%	25	12.8%	19	6.9%	15	6.8%	18	5.6%	
	Competitive field	3	21.4%	5	14.7%	5	6.4%	17	8.7%	16	5.8%	12	5.4%	19	5.9%	
	Research opportunities	3	21.4%	4	11.8%	5	6.4%	17	8.7%	15	5.4%	9	4.1%	20	6.2%	
	Others	0	0.0%	1	2.9%	4	5.1%	13	6.6%	14	5.1%	4	1.8%	18	5.6%	
	The prestige associated with the specialty	0	0.0%	1	2.9%	4	5.1%	9	4.6%	9	3.2%	13	5.9%	6	1.9%	
	Having a role-model	0	0.0%	2	5.9%	2	2.6%	4	2.0%	8	2.9%	12	5.4%	14	4.4%	
	Variety of the cases and population character	0	0.0%	0	0.0%	1	1.3%	4	2.0%	7	2.5%	8	3.6%	10	3.1%	
	The geographic location of the training centers	0	0.0%	0	0.0%	1	1.3%	3	1.5%	2	0.7%	4	1.8%	3	0.9%	
Most deterring factor	Risk	3	21.4%	6	17.6%	19	24.4%	41	20.9%	54	19.5%	35	15.8%	47	14.6%	<0.001*
	Lifestyle, work-life balance	1	7.1%	4	11.8%	12	15.4%	28	14.3%	40	14.4%	45	20.4%	69	21.5%	
	Stress	1	7.1%	3	8.8%	3	3.8%	16	8.2%	23	8.3%	26	11.8%	43	13.4%	
	Other career interests	1	7.1%	3	8.8%	8	10.3%	30	15.3%	30	10.8%	16	7.2%	27	8.4%	
	Long training time	0	0.0%	5	14.7%	8	10.3%	17	8.7%	33	11.9%	30	13.6%	22	6.9%	
	Competitive field	0	0.0%	4	11.8%	6	7.7%	14	7.1%	19	6.9%	25	11.3%	19	5.9%	
	Difficulty/neurophobia	1	7.1%	1	2.9%	7	9.0%	10	5.1%	23	8.3%	11	5.0%	20	6.2%	
	Male dominated	4	28.6%	5	14.7%	1	1.3%	14	7.1%	13	4.7%	7	3.2%	22	6.9%	
	Others	0	0.0%	2	5.9%	2	2.6%	6	3.1%	11	4.0%	3	1.4%	15	4.7%	
	Few trainings centers	2	14.3%	0	0.0%	2	2.6%	4	2.0%	6	2.2%	13	5.9%	10	3.1%	
	Practical aspect, long surgeries	0	0.0%	0	0.0%	3	3.8%	8	4.1%	13	4.7%	4	1.8%	6	1.9%	
	Complex patients	1	7.1%	0	0.0%	2	2.6%	5	2.6%	3	1.1%	5	2.3%	13	4.0%	
	Limited job opportunity	0	0.0%	1	2.9%	2	2.6%	1	0.5%	6	2.2%	1	0.5%	3	0.9%	
	Income	0	0.0%	0	0.0%	3	3.8%	2	1.0%	3	1.1%	0	0.0%	5	1.6%	
^F^Fischer's Exact Test *P<0.05, Significant																

Most attractive factors: While there are no statistically significant findings, some interesting trends emerge. Students with CGPAs in the range of 3.0-3.49 and 4.0-4.49 show a relatively higher interest in factors such as "interested in neuroscience," "impact on patients-rewarding," and "challenging specialty."

Most deterring factors: In contrast, the "risk" associated with neurosurgery appears to be the most deterring factor for students across different CGPA ranges. This factor is particularly concerning for students with CGPAs below 2.5. Additionally, "lifestyle, work-life balance," "stress," and "other career interests" also show increased concerns among students with CGPAs in the lower ranges (p=<0.001*).

Region

Students hailing from the eastern region displayed the highest attraction, 39 (20.4%), to the notion of being "interested in neuroscience," which was significantly more pronounced compared to their peers in the southern region, 16 (11.6%), and western region, 42 (19.4%). Furthermore, the perception of a "rewarding" impact on patients appeared more enticing to students in the eastern region, 32 (16.8%), and central region, 43 (13.6%), significantly surpassing the interest levels in the northern region, 25 (8.9%), and southern region, 16 (11.6%). Additionally, students residing in the northern region found the "challenging specialty" more appealing, 39 (13.9%), indicating a regional variation in preferences. (p = 0.011*) (Table 8).

**Table 8:** Association of the most attractive and deterring factors with region

		Region										
		Central		Eastern		Northern		Southern		Western		P value^F^
		n	Column percentage %	n	Column percentage %	n	Column percentage %	n	Column percentage %	n	Column percentage %	
Most attractive factor	Interested in neuroscience	49	15.5%	39	20.4%	49	17.5%	16	11.6%	42	19.4%	0.011*
	Impact on patients “rewarding”	43	13.6%	32	16.8%	25	8.9%	16	11.6%	27	12.5%	
	Challenging specialty	41	13.0%	22	11.5%	39	13.9%	8	5.8%	22	10.2%	
	Practical aspect, surgical techniques or high skill required	36	11.4%	23	12.0%	31	11.1%	17	12.3%	20	9.3%	
	Income	28	8.9%	21	11.0%	38	13.6%	15	10.9%	22	10.2%	
	Innovation/technology	18	5.7%	13	6.8%	15	5.4%	25	18.1%	18	8.3%	
	Competitive field	25	7.9%	6	3.1%	22	7.9%	11	8.0%	13	6.0%	
	Research opportunities	23	7.3%	12	6.3%	14	5.0%	11	8.0%	13	6.0%	
	Others	16	5.1%	6	3.1%	12	4.3%	7	5.1%	13	6.0%	
	The prestige associated with the specialty	13	4.1%	2	1.0%	15	5.4%	2	1.4%	10	4.6%	
	Having a role-model	10	3.2%	8	4.2%	14	5.0%	2	1.4%	8	3.7%	
	Variety of the cases and population character	7	2.2%	6	3.1%	6	2.1%	5	3.6%	6	2.8%	
	The geographic location of the training centers	7	2.2%	1	0.5%	0	0.0%	3	2.2%	2	0.9%	
Most deterring Factor	Risk	58	18.4%	39	20.4%	41	14.6%	27	19.6%	40	18.5%	0.002*
	Lifestyle, work-life balance	69	21.8%	36	18.8%	39	13.9%	22	15.9%	33	15.3%	
	Stress	22	7.0%	32	16.8%	28	10.0%	10	7.2%	23	10.6%	
	Other career interests	21	6.6%	19	9.9%	35	12.5%	16	11.6%	24	11.1%	
	Long training time	35	11.1%	19	9.9%	24	8.6%	17	12.3%	20	9.3%	
	Competitive field	23	7.3%	4	2.1%	40	14.3%	9	6.5%	11	5.1%	
	Difficulty/neurophobia	23	7.3%	9	4.7%	16	5.7%	8	5.8%	17	7.9%	
	Male-dominated	19	6.0%	7	3.7%	20	7.1%	6	4.3%	14	6.5%	
	Others	13	4.1%	3	1.6%	5	1.8%	6	4.3%	12	5.6%	
	Few trainings centers	9	2.8%	3	1.6%	15	5.4%	4	2.9%	6	2.8%	
	Practical aspect, long surgeries	8	2.5%	6	3.1%	6	2.1%	7	5.1%	7	3.2%	
	Complex patients	9	2.8%	6	3.1%	5	1.8%	4	2.9%	5	2.3%	
	Limited job opportunity	3	0.9%	5	2.6%	4	1.4%	1	0.7%	1	0.5%	
	Income	4	1.3%	3	1.6%	2	0.7%	1	0.7%	3	1.4%	
*P<0.05, Significant ^F^Fischer's Exact Test												

The factor of "risk" associated with neurosurgery proved to be significantly more deterring to students in the central region, 58 (18.4%), and eastern region, 39 (20.4%), as opposed to those in other regions. Moreover, the desire for a "lifestyle and work-life balance" was notably higher among students in the central region, 69 (21.8%), and eastern region, 36 (18.8%), significantly surpassing the interest levels of their peers in the northern region, 39 (13.9%), and southern region, 22 (15.9%). The perception of "stress" was particularly pronounced among students in the eastern region, 32 (16.8%), significantly exceeding that of students in other regions. Of students originating from the northern region, 40 (14.3%), expressed more deterrence due to the "competitive field" (p = 0.002*).

## Discussion

Choosing a future career is essential for medical students, and this must be accomplished during their university days. Neurosurgery is one of the most recognized surgical specialties. Nevertheless, as indicated by observations throughout time, it is not among Saudi Arabia's top selections for medical students [13]. Highly influenced by individual preferences and exposure to the work environments, factors that are considered by medical students to pursue or deter this specialty are a challenging specialty, impact on patients' "rewarding," interest in neuroscience, risk, lifestyle, work-life balance, and stress. As a constantly developing specialty with quick advancements in surgical procedures and management, neurosurgery sets itself apart among the medical specialties [2].

Every year, five million critical neurosurgical cases in countries with middle and low incomes are ignored. On a local basis, a report from 2019 estimated that the number of neurosurgeons is approximately 1/250,000 of the population in the Kingdom of Saudi Arabia [2]. An expanding population made it necessary for the development of a program for neurosurgery training [6]. The average age observed in our study was 21.7 years, with 59.9% female participation. Predominant female participation was observed in many other studies [13-17]. However, a predominant male participation was observed in some other studies [2,18]. A predominant mean age between 21 to 24 years of age has been seen by most of the authors [12,15,17-18]. Most participants in our study were second-year students, and the maximum participation was from the central region.

A Nigerian study done among final-year medical students revealed that 85.2% of their participants reported unpleasant experiences with neurosurgical postings, which included early and long ward rounds and long duration of ward sessions, and only 12.5% of them were satisfied with their neurosurgical postings [19]. According to a study reported in 2022, only 88 out of 278 medical students with formal training in neurosurgery expressed definite interest in a career in neurosurgery, with the remaining students reporting no possible interest [18]. Among the participants in our study, approximately half expressed an interest in pursuing a career in neurosurgery, and a majority claimed to understand what such a career entails. However, a lower percentage feels confident in their understanding of the requirements to secure a training position in neurosurgery. Some studies have reported a higher percentage (>70%) of students expressing their interest in pursuing a career in neurosurgery [14,15].

It is important to note that some studies have identified an exception to the existing literature. Zoli M. et al. observed in their study that 101 students (64.7%) thought neurosurgery may be an interesting field to pursue in the future [20]. Akhigbe et al. stated that 78% of the students would consider neurosurgery as a future career [15]. Al Sharqi Ali found that 71.7% of their participants would be interested in neurosurgery as a future career [14]. Indeed, exposure to any clinical specialty throughout medical school has a significant impact on the student's career decisions. Exposure to the issues and unique challenges that each specialty provides, as well as the work environment that is typical of each specialty [2].

The decision of students to pursue a career in a specialty is influenced by many factors, according to the literature: exposure to that specialty during clinical rotations, spending time in operating rooms, having access to and communication with surgeons, having professional relationships, and mentor-mentee relationships [21]. To motivate the upcoming generation of neurosurgeons and to guarantee that the specialty draws the top individuals who would positively impact the field, enough exposure to neurosurgery is essential. One of the biggest problems that this field has is the shortage of staff, so by enhancing exposure to this field, we can address this deficit [22].

Neurosurgery tends to be introduced to medical students late in their clinical years, possibly when they have already made up their decisions about their future careers. According to several studies, medical students stay away from a career in neurosurgery because their earlier clinical exposure was confined to other surgical specialties [14,15,23]. Regarding work-life balance, a study done by Wang et al. stated that reduced job satisfaction was reported in 18.5% of females, and 20.4% said they would not want to pursue neurosurgery again due to a lack of protected personal time [24]. In contrast, in a Japanese study assessing women neurosurgeons, 83.8% of participants were satisfied with their jobs, 65.7% with their work timeline, and 51.9% with their work-life balance [25].

The top three attractive factors about neurosurgery in our study were the challenging nature of the specialty (50.9%), interest in neuroscience (45.5%), and the practical aspects and high skill requirements of the specialty (42.8%). Alghamdi et al., in their study, observed that positive impact on patients' rewarding, income, and interest in neuroscience were the top three attractive factors [2]. Al Sharqi Ali et al. stated that the factors that may improve medical students' and interns’ education in neurosurgery are the presence of mentors in neurosurgery, adding neurosurgery rotation, and the huge prestige and income associated with the specialty [14]. A study done by Kaliyadan et al. reported that about 55% agreed that neurosurgery being a challenging specialty would positively influence their choice as an attractive factor [26].

The top three deterring factors about neurosurgery in our study were stress (50.3%), long training duration (50.0%), and concerns related to lifestyle and work-life balance (49.5%). Alghamdi et al., in their study, observed that stress, difficulty/neurophobia, and risk are the top three deterring factors [2]. Al Sharqi Ali et al. considered the drawbacks to be the challenging nature of neurosurgical cases, the absence of a neurosurgery residency program, and the long training period of the specialty [14].

This analysis did not reveal any significant age variation for the most attractive factors, whereas, among the deterring factors, only the perception of a few training centers was associated with older participants. Alghamdi et al. found that younger students were significantly more interested than older students and interns [2]. Similarly, Alnaami I. et al. found that 40% of students were interested, whereas only 26% of interns were interested in pursuing a career in neurosurgery [13].

This analysis also revealed statistically significant gender differences in factors. Specifically, a higher percentage of females found "interested in neuroscience" to be the most attractive factor compared to males (18.2% vs. 15.5%). Regarding deterring factors, a greater percentage of males found "risk" as the most deterring factor compared to females (19.2% vs. 17.1%). Efe IE et al. stated that interest in neurosurgery was significantly greater among male students. They observed that the female students were attracted to the specialty for the delicate manual work, whereas the challenging and rapidly evolving nature of the specialty was an attractive factor for the male students. Furthermore, the lack of work-life balance was a deterrent factor, with females finding it not a family-friendly specialty and males considering it unpleasant and too hierarchical [16]. Alghamdi et al. observed that males and females are not equally interested in neurosurgery, with females showing more interest as compared to males [2]. Roy B et al. stated that poor quality of life and excessive clinical activities were seen as drawbacks by males, whereas workload was the most important factor for females [23].

The analysis showed no statistically significant differences in the most attractive or deterring factors among different educational or clinical levels. These findings suggest that most factors are consistently attractive or deterring across different educational and clinical levels, emphasizing the stability of these perceptions throughout medical education. However, Alghamdi et al. found that the education level of participants affected their interest in neurosurgery significantly, and an increased educational level was found to decrease the level of interest in the field [2]. Regarding the association of academic CGPGA with the most attractive factors, no statistically significant findings were observed. However, about the most deterring factors, the "risk" associated with neurosurgery was the most deterring factor for students across different CGPA ranges, particularly for students with CGPAs below 2.5. We also observed that "lifestyle, work-life balance," "stress," and "other career interests" show significantly increased concerns among students with CGPAs in the lower ranges.

Students from the eastern region displayed the highest attraction to "interested in neuroscience" and the perception of a "rewarding" impact on patients, whereas students residing in the northern region found the "challenging specialty" more appealing. The factors of "risk" associated with neurosurgery and "lifestyle and work-life balance" proved to be significantly more deterring to students in the central and eastern regions. The perception of "stress" was particularly pronounced among students in the eastern region, significantly exceeding that of students in other regions. However, students from the northern region expressed more deterrence due to the "competitive field." We did not encounter any studies that compared the different regions of the Kingdom with respect to the perceptions of students toward neurosurgery.

Limitations of the study

While this study offers a comprehensive overview of factors affecting the choice of neurosurgery as a future career, it is essential to mention its inherent limitations. The cross-sectional nature restricts the determination of causality, and reliance on self-reported data could lead to biases.

## Conclusions

Neurosurgery, a challenging and ever-emerging specialty, represents one of the most recognized specialties among medical students. Approximately half of the participants in our study expressed their interest in pursuing a career in neurosurgery and found it appealing due to its challenging nature. However, deterring factors like stress, long training duration, and concerns related to lifestyle and work-life balance were also observed in around half of the participants. Clinical phase students and interns showed the least interest, whereas pre-clinical students, students with low GPAs, and students exposed to neurosurgical education exhibited the highest interest. Participants from the northern region showed more interest in the field.

We found no significant age variation in the most attractive factors; however, significant gender differences in attractive and deterrent factors were observed. The "risk" associated with neurosurgery was the most deterring factor for students across different CGPA ranges and students from the eastern and central areas. Our findings suggest that most factors are consistently attractive or deterring across different educational and clinical levels, emphasizing the stability of these perceptions throughout medical education. We propose innovative educational initiatives with increased faculty participation to implement the curricula with early exposure of students to neurosurgery.
